# Bacterial epiglottitis superimposed on oropharyngeal cancer: A case report

**DOI:** 10.1002/cnr2.1783

**Published:** 2023-01-23

**Authors:** Michael C. Larkins, David B. Meyer, Kenneth Lowery, Rachel L. Speicher, Brian N. Brodish, Andrew W. Ju

**Affiliations:** ^1^ Brody School of Medicine East Carolina University Greenville North Carolina USA; ^2^ East Carolina Anesthesia Associates Greenville North Carolina USA; ^3^ Eastern Carolina ENT Head and Neck Surgery Greenville North Carolina USA; ^4^ Department of Radiation Oncology East Carolina University Brody School of Medicine Greenville North Carolina USA

**Keywords:** airway‐obstructing tumor, emergent tracheostomy, epiglottitis, oropharyngeal cancer, recurrent airway infection

## Abstract

**Background:**

Patients undergoing chemotherapy and radiotherapy are placed in an immunocompromised state worth consideration in the event of potential airway compromise, especially when superimposed on an airway‐obstructing tumor. We report a case of bacterial epiglottitis in a patient with active oropharyngeal cancer (OPC), who presented in such a way that an infectious etiology was not initially considered in the patient's care. To our knowledge, such a circumstance has not been reported in the literature.

**Case:**

Here, we report a case of a 68‐year‐old male with advanced‐stage OPC who developed respiratory distress and underwent emergent tracheostomy. The patient was diagnosed postoperatively with *Haemophilus influenza* and *Pseudomonas aerugeniosa*. Following antibiotic treatment, the patient recovered to the point in which he could then undergo concomitant chemoradiation. The patient later had a recurrence of *P. aerugeniosa* during their radiotherapy that was also treated with antibiotics. The patient experienced continued symptoms related to their OPC and underwent pharyngectomy. Despite the initial success of this procedure, the patient experienced tumor recurrence and succumbed to his disease.

**Conclusion:**

This case underscores the importance of considering multiple etiologies concerning airway compromise, as the consequence of delayed cancer treatment may be loss of local cancer control.

## 1 BACKGROUND

Head and neck cancer accounts for 3% of all cancers in the United States, with the overall five‐year survival rate in oropharyngeal carcinoma (OPC) being 60% and with HPV‐positive OPC showing a better prognosis and increased treatment response compared to HPV‐negative cancer.^1^ Head and neck cancers can lead to an immunodeficient state predisposing patients to upper respiratory infections such as epiglottitis, which can acutely threaten a patient's life and may delay treatment, ultimately leading to cancer recurrence; more than 50% of all adults with epiglottitis have an underlying comorbidity that predisposes them to epiglottitis, and due to the widespread vaccination of children, adults are now the most likely population to present with this infection.^2^, ^3^, ^4^ The majority of epiglottitis cases are bacterial, though viral, fungal, and noninfectious epiglottitis have all been reported.^5^, ^6^, ^7^ Patients with epiglottitis often require aggressive treatment and intensive care due to the risk of airway compromise, making early diagnosis critical. This care can include intubation or tracheostomy creation; however, the requirement to intubate a patient may be avoidable with prompt diagnosis and treatment with intravenous (IV) antibiotics and corticosteroids.^8^, ^9^ There is a dearth of literature available concerning both epiglottitis and oropharyngeal cancer, especially regarding the role of epiglottitis as a confounding factor in rapid airway evaluation. Cases of epiglottitis in patients undergoing chemotherapy^10^ and in which tumors of the airway can present in a similar manner to acute epiglottitis^11^ have both been reported, but no specific instance of epiglottitis superimposed on oropharyngeal cancer has been reported to date.

## 2 CASE

**FIGURE 1:**
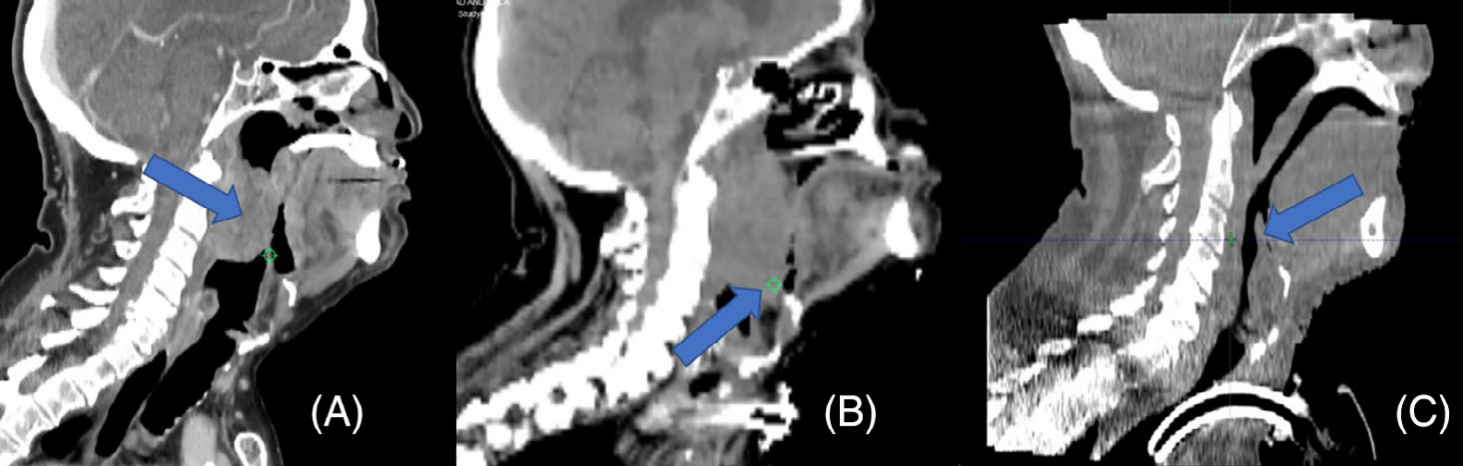
A—Sagittal CT view of the tumor and normal epiglottis on initial presentation to the Emergency Department, with arrow pointing to patient's tumor. B—Sagittal view of the tumor and enlarged epiglottis now contacting the tumor and causing airway obstruction, 14 days after admission (arrow points to tumor contacting epiglottis). C—Sagittal view showing an excellent tumor response and resolution of epiglottis by the last day of radiotherapy (arrow points to normal‐sized epiglottis, no longer in contact with tumor)

A 68‐year‐old man with recently diagnosed Stage IVA, cT4a cN2c cM0 moderately differentiated, p16 negative oropharyngeal squamous cell carcinoma of the posterior pharyngeal wall presented to the Emergency Department (ED) of ECU Health Medical Center in Greenville, NC in May 2021 with throat pain and dysphagia. The patient's regional oncology clinic had concerns for airway compromise, and it was noted that the patient's radiotherapy had been delayed as the patient required dental clearance and could not lay flat without desaturation. The patient's initial physical exam revealed a visible mass raising concern for potential airway compromise. Computed tomography (CT) imaging of the neck was obtained (Figure 1A) showing a large occluding mass in the oropharynx; the radiologist's impression described an increased size of the patient's pharyngeal mass with moderate narrowing of the oropharyngeal airway. Shortly after admission, the patient became somnolent and tachycardic, and saw a drop in oxygen saturation to 86%; otolaryngology was then consulted.

On assessment by the otolaryngology service, the patient was noted as being lethargic; their mouth was open, and tongue was protruding, and the patient was using their abdominal, neck, and shoulder accessory muscles to breathe. Lung sounds were noted as diminished. It was also noted that the patient had a high‐flow nasal cannula in place set to 15 L. The patient aroused to verbal engagement but was difficult to understand due to their voice being raspy. Given the patient's known airway‐obstructing tumor with minimal area of airway patency, the otolaryngology service considered the patient to be a difficult airway and decided to perform an emergent tracheostomy in the operating room. No endoscopy was performed, as this assessment was considered likely to confirm suspicions that the patient's airway was minimally patent.

**FIGURE 2:**
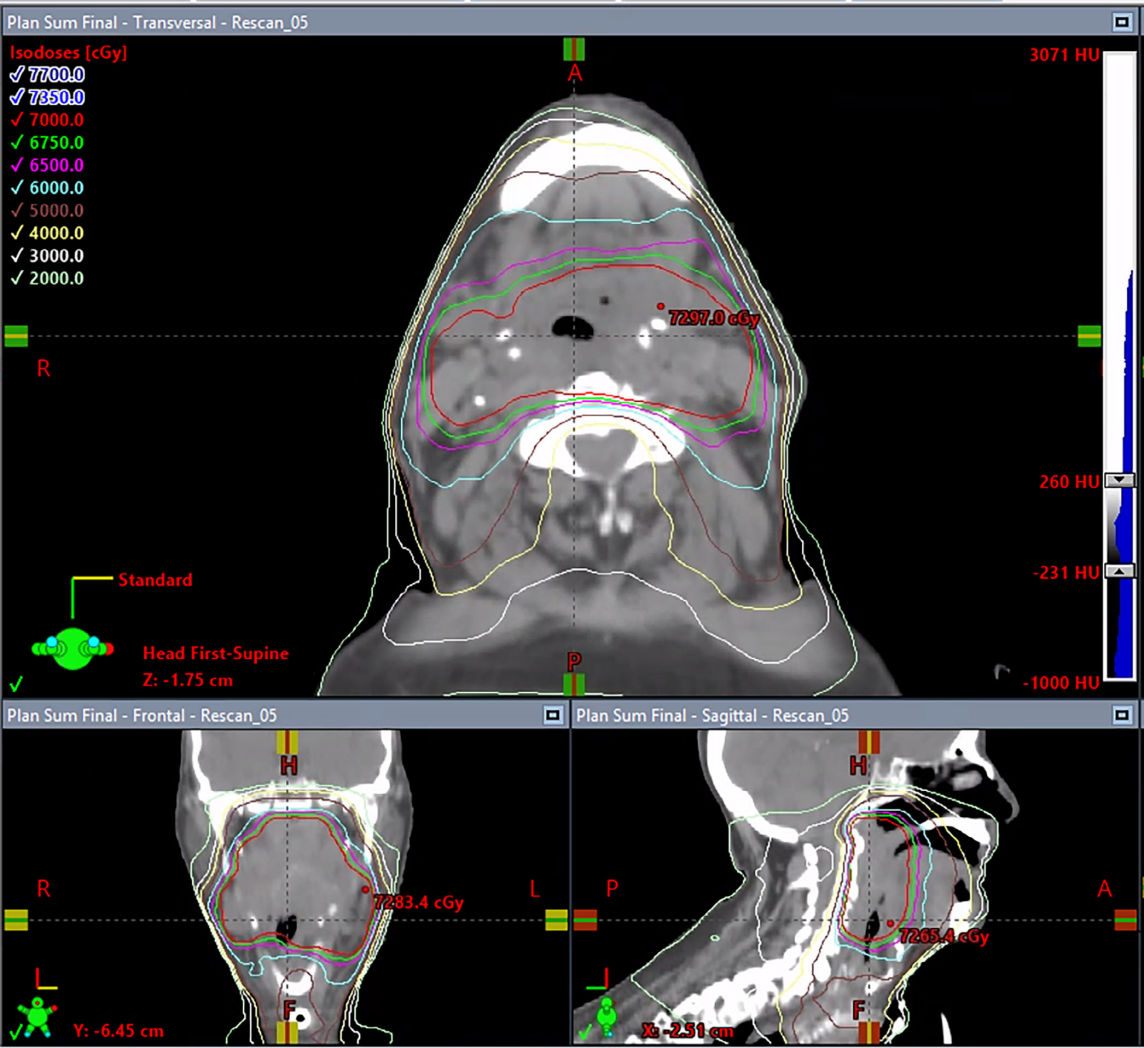
The patient's dose distribution mapping in three views, showing both an oropharyngeal tumor and epiglottitis. Note the inflamed epiglottitis contacting the tumor

The patient was placed on the operating room table in the supine position. After general anesthesia was induced and the neck was sterilely prepped and draped, an incision was performed in the inferior midline of the patient's neck. Dissection was carried out to the median raphe of the strap muscles, which were subsequently dissected and retracted to expose the upper trachea. The thyroid isthmus was divided vertically via electrocautery, and a cuffed Shiley tracheostomy tube was inserted between the second and third cartilaginous rings, which was then secured via 00 Prolene sutures. The procedure was considered relatively straightforward by the otolaryngology service, as the tracheostomy site was distal to the patient's airway‐obstructing tumor and was accomplished in approximately 30 min. The patient remained hemodynamically stable throughout the procedure. The patient was transported to the intensive care unit (ICU), and later a respiratory panel taken on Day 5 of his hospital admission grew *P. aeruginosa* and *H. influenzae* on culture. The patient required an urgent start of radiation due to tumor bleeding, with planned initial doses of 600 cGy delivered in two fractions to the head and neck, and 400 cGy delivered in two fractions to the supraclavicular regions. The patient's radiotherapy planning CT imaging reflected a diagnosis of epiglottitis contributing to airway obstruction (Figure 1B). He began treatment with 3.375 g of IV piperacillin/tazobactam every 8 hours for 1 week with good response. The patient went on to complete their definitive radiotherapy for oropharyngeal cancer (at 200 cGy/fraction for 22 fractions with an additional 10 fraction boost; see Figure 2 for dose distribution mapping) despite multiple treatment delays due to the patient's requirement for sedation, ventilation, and inpatient resource coordination, with an excellent response by the last day of radiotherapy (Figure 1C). The patient's tracheostomy was subsequently closed with adequate tumor shrinkage. He was discharged to a skilled nursing facility (SNF) in good condition.

**FIGURE 3:**
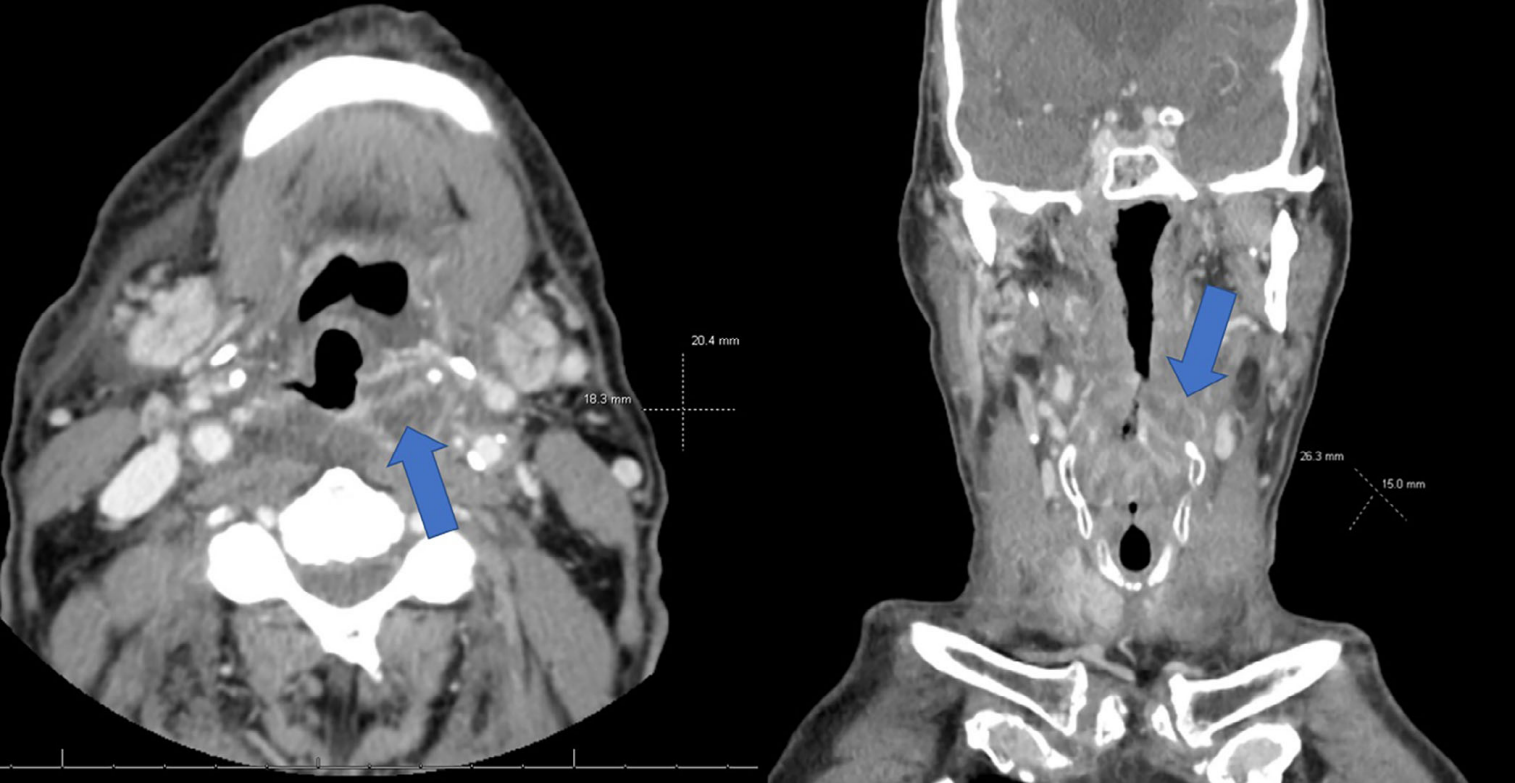
Head and neck CT axial (left) and coronal (right) views showing the patient's recurrent oropharyngeal mass, with blue arrows pointing directly to the mass. Measurements to the right of each view show the approximate cross‐sectional area of the mass. This lesion was considered to be resectable based on imaging.

**FIGURE 4:**
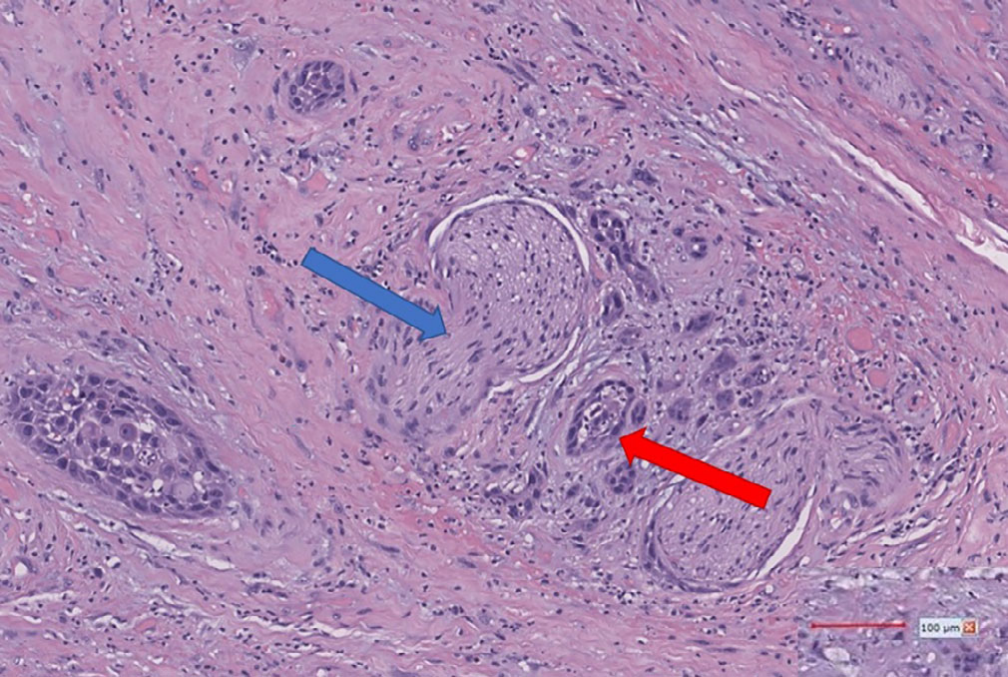
Histopathological examination of the patient's malignant neoplasm, with demonstrated perineural invasion with atypical keratinized squamous cells (blue arrow) and foreign body giant cell reaction (red arrow). Samples were obtained during partial pharyngectomy with left modified neck dissection for recurrent squamous cell carcinoma (10X magnification).

During radiation follow‐up about 10 weeks after discharge, the patient reported increasing dysphagia and odynophagia along with chills and hoarseness. Follow‐up soft tissue neck CT revealed a mucosal mass at the left piriform sinus, at the inferior margin of the previously noted oropharyngeal mass (see Figure 3). Laryngoscopy revealed widespread, generalized edema of the oropharynx and larynx, with an ulcerated lesion located posterior to the hypopharyngeal wall centered just left of midline. Given that the patient had undergone both radiotherapy and chemotherapy previously with minimal long‐term improvement in his symptoms and the patient's lesion appeared resectable radiographically, surgery as salvage therapy was considered the patient's last option for symptomatic relief. The patient underwent successful partial pharyngectomy with left modified neck dissection 5 months after completion of radiotherapy. The patient ultimately tolerated the procedure; resection specimen (see Figure 4) showed TNM staging: pT2 pN0 pM0 moderately differentiated. The patient's pathology report noted a residual invasive moderately differentiated squamous cell carcinoma forming a 3.0 × 2.7 × 2.0 cm mass. The tumor did not involve the hyoid bone or thyroid cartilage. Lymphovascular invasion was not identified and perineural invasion was present (see Figure 4). PD‐L1 was expressed with a combined positive score of 70; p16 study was negative.

Following surgery, the patient was stable and was planned to be kept under close surveillance at a SNF. Due to transportation difficulties the patient was unable to make his follow‐up appointments for reassessment. Five months later, the patient presented to outpatient otolaryngology clinic with increased throat pain and dysphagia concerning for recurrence of disease; oropharynx biopsy revealed a malignant neoplasm with infiltrating nests of atypical keratinized squamous cells. The patient was placed under palliative care measures and ultimately succumbed to his disease 2 weeks later.

## 3 DISCUSSION

Cancer and its treatment modalities (chemotherapy, radiotherapy, and surgery) all increase the risk for infection in patients.^12^, ^13^ Airway considerations for oropharyngeal cancer often involve the cancer itself, but perioperative assessment should not mistake the obvious pharyngeal mass and fail to consider the potential of other etiologies of airway compromise in such patients such as infection.^14^ The patient in this case report developed airway obstruction secondary to epiglottitis in the context of oropharyngeal cancer, visible in Figure 1 B, and presented in such a way that infection was low on the differential. It was not until the patient presented with infection symptoms and tested positive a second time for *P. aeruginosa* that a diagnosis of epiglottitis was considered. This diagnosis is usually readily established by the visualization of the inflamed supraglottic area on direct laryngoscopy or CT imaging and is important to recognize early due to the high risk of sudden airway obstruction in adults, as seen in this patient.^9^, ^15^ However, it is important to note that the diagnosis of epiglottitis is much less straightforward in the setting of tumor progression during active malignancy. The narrowing of the patient's airway was initially attributed solely to the increasing size of the patient's oropharyngeal cancer. Cases of epiglottitis in immunocompromised patients within the context of cancer have been reported in the literature, but no case to the authors' knowledge has reported on the potential of bacterial epiglottitis to be missed due to obfuscation from an airway‐occluding mass.^16^, ^17^

Pharyngeal masses present a potentially difficult challenge for airway management. Complete airway obstruction resulting in loss of access to the airway could prove disastrous in an emergent situation. Common risks that pharyngeal masses pose to airway integrity include the risk of bleeding, local tumor invasion into supportive structures leading to airway obstruction, and from the bulk of the tumor directly. The literature describes a systematic approach in the management of patients with airway masses.^18^ It should be noted that history of head and neck radiotherapy is not necessarily associated with additional risk of intubation, and cancer site can dictate the need for and success of intubation or tracheostomy.^19^, ^20^

## 4 CONCLUSION

Literature suggests that early diagnosis permitting treatment of epiglottitis with IV antibiotics and corticosteroids decreases the need for airway intervention and as such would decrease the risks associated with tracheostomies.^9^ However, there is a dearth of literature reporting the diagnosis and treatment of epiglottitis among head and neck cancer patients with active malignancy or an airway‐occluding mass. It is therefore recommended for cases where upper airway obstruction and pharyngeal tumor are evident, to include a rapid evaluation of a compromised airway. Additionally, we suggest that a broad differential be considered to include infectious etiologies superimposed on active malignancy. The case we presented suggests that a rapid diagnosis of epiglottitis contributing to airway compromise may have directed treatment, potentially avoiding the need for an emergent tracheostomy. The delay in onset of radiotherapy may have contributed to the patient's recurrence of oropharyngeal cancer.

## AUTHOR CONTRIBUTIONS

**Michael Larkins:** Investigation (lead); methodology (lead); project administration (lead); writing – original draft (lead); writing – review and editing (lead). **David Meyer:** Project administration (supporting); writing – original draft (supporting); writing – review and editing (supporting). **Kenneth Lowery II:** Project administration (supporting); writing – original draft (supporting). **Rachel Speicher:** Project administration (supporting); supervision (supporting); writing – review and editing (supporting). **Brian Brodish:** Project administration (supporting); supervision (supporting); writing – original draft (supporting); writing – review and editing (supporting). **Andrew Ju:** Conceptualization (lead); project administration (supporting); resources (lead); supervision (lead); writing – original draft (supporting); writing – review and editing (supporting).

## FUNDING INFORMATION

The authors received no financial support for the research, authorship, and/or publication of this article.

## CONFLICT OF INTEREST

The authors have stated explicitly that there are no conflicts of interest in connection with this article.

## ETHICAL STATEMENT

Written informed consent was obtained from the patient for publication of case details, including imaging.

## Data Availability

The data that support the findings of this study are available from the corresponding author upon reasonable request.
